# Should MMMT still be treated with adjuvant taxane-based combination chemotherapy?

**DOI:** 10.1007/s00432-019-03091-y

**Published:** 2020-01-28

**Authors:** Viola Heinzelmann-Schwarz, André B. Kind, Marcus Vetter, Kenneth Russell, Siti Omar, Andreas Schoetzau, Kerstin Hoeck, Daniel Fink, Michael L. Friedlander, Neville F. Hacker

**Affiliations:** 1 Gynecological Cancer Centre, Royal Hospital for Women, School of Women’s and Children’s Health, University of New South Wales, Sydney, NSW 2031 Australia; 2 Gynecological Cancer Centre, Hospital for Women, University of Basel, Spitalstrasse 21, 4021 Basel, Switzerland; 3 Caris Life Sciences, Basel, Switzerland; 4 Department of Gynecology, University Hospital Zurich, Zurich, Switzerland; 5 Medical Oncology, Prince of Wales Hospital, Prince of Wales Clinical School, University of New South Wales, Sydney, NSW 2031 Australia

**Keywords:** Carcinosarcoma, Epirubicin, Ifosfamide, Paclitaxel, Ovarian cancer, Endometrial cancer

## Abstract

**Background:**

Malignant mixed Mullerian tumors of endometrial (MMMT-E) and ovarian (MMMT-O) origin are associated with poor prognosis. Suggestively epithelial-driven tumors, their treatment has shifted from anthracycline or ifosfamide-based towards taxane-based chemotherapy. It remains unclear whether this change associates with better outcomes.

**Patients and methods:**

A conjoined Australian and Swiss patient cohort of MMMT-E (*N* = 103) and MMMT-O (*N* = 17) was compared to patients with adenocarcinoma of the endometrium (EC, *N* = 172) and ovary (OC, *N* = 189). Clinicopathological characteristics, FIGO stage, first-line treatment, and patient outcomes were analyzed. The generated hypothesis was verified in an US-American cohort with high-grade serous ovarian cancer (HGSOC, *N* = 1290) and MMMT-O (*N* = 450) using immunohistochemistry and next-generation sequencing.

**Results:**

Early stage I/II MMMT-E showed a survival plateau after 2.5 years, with no recurrence or death observed afterwards. Relapse-free survival was significantly worse in MMMT-E treated with platinum/taxanes (*P* = 0.024) compared to non-taxane regimen. Hypothesizing that also MMMT-O might benefit from an adjuvant non-paclitaxel regimen, a second independent cohort of MMMT-O and HGSOC patients was examined. p53 mutations dominated in both cancers with comparable frequency. PI3KCA and KRAS mutations were less frequent: they were more frequent in MMMT-O than in HGSOC (*P* = 0.015 and *P* = 0.018, respectively). MMMT-O responded better to a combination of carboplatin with anthracyclines than with taxanes (73.9% vs. 39.4%).

**Conclusion:**

Early stage I/II MMMT-E patients have excellent prognosis if no recurrence has appeared within the first 2.5 years. In MMMT-E, platinum/anthracycline or ifosfamide regimen associated with better outcomes than platinum/taxanes regimens. This might also apply to MMMT-O.

**Electronic supplementary material:**

The online version of this article (10.1007/s00432-019-03091-y) contains supplementary material, which is available to authorized users.

## Introduction

Carcinosarcoma or malignant mixed Mullerian tumors (MMMT) are biphasic tumors that contain malignant mesodermal and epithelial components, in contrast to sarcomas, which contain exclusively malignant mesodermal elements (Kernochan and Garcia 2009). Typically, the metastatic sites mainly consist of carcinoma elements (Silverberg et al. 1990). Due to their rareness, there are no large epidemiological studies available and data are in general inconclusive. It has been proposed that the epithelial and sarcomatous elements develop from the same stem cell, with the carcinoma component being undifferentiated and of a specific histotype, whilst the sarcomatous component shows a mixed homologous appearance (McCluggage 2002; Gorai et al. 1997). Relapse-free and overall survival in MMMT-E is poor, and treatment failure at the time of recurrence appears to be at distant sites (Gadducci et al. 2002; Gonzalez Bosquet et al. 2010; Yamada et al. 2000; Manolitsas et al. 2001; Vorgias and Fotiou 2010). Surgical therapy is the initial treatment modality and necessitates hysterectomy, bilateral salpingo-oophorectomy and at least pelvic lymphadenectomy (NCCN 2001). If metastatic disease is present, there is a need for full cytoreduction, which can be achieved in 57% of patients, resulting in a significant improvement of median overall survival (OS 53.3 vs. 8.6 months) (Tanner et al. 2011).

The benefit of chemotherapy for MMMT-E has been clearly shown, with active substances being cisplatin, carboplatin, ifosfamide, anthracyclines and paclitaxel (Sutton et al. 2000, 2005). It has been proposed that FIGO Stage I patients should be treated with 4 cycles and Stage II–IV patients with 6 cycles of carboplatin and epirubicin (Manolitsas et al. 2001; Berton-Rigaud et al. 2014; Wolfson et al. 2007). A recent Cochrane review including 579 patients from two randomized studies compared adjuvant radiotherapy with combination chemotherapy (ifosfamide with paclitaxel versus ifosfamide alone) in recurrent Stage III/IV MMMT-E, finding an improved OS in the combination chemotherapy treatment arms (Sutton et al. 2000; Homesley et al. 2009; Galaal et al. 2011). The promising survival data from various studies and the reduced toxicity found in the carboplatin and paclitaxel combination treatments which are predominantly used in endometrial and ovarian adenocarcinoma, led several institutions, including our own, to change the chemotherapy regimen in MMMT-E to this more tolerable combination. However, there is no established consensus for therapeutic management in this patient group (Berton-Rigaud et al. 2014; NCCN 2001). With only a few prospective randomized controlled trials reported in MMMT-E, the optimal chemotherapy modality is yet to be determined, particularly in view of new targeted therapies.

Therefore, this study retrospectively analyzed the outcomes of a large cohort of MMMT-E to evaluate the response to various kind of adjuvant chemotherapies.

## Materials and methods

### Cohort description and outcome analysis

We reviewed all patients with MMMT-E treated with an adjuvant platinum-based chemotherapy in the Gynecological Cancer Centre of the Royal Hospital for Women in Sydney, Australia and the Gynecological Cancer Centers of the University Hospitals Zurich and Basel, Switzerland between 1988 and 2014 (*N* = 103). For each case, at least 1 control of EC (*N* = 172) and OC (*N* = 189) undergoing adjuvant platinum-based therapy was matched by age and FIGO Stage from the equivalent databases available. Data of cases and controls were obtained using paper and electronic patient records, as available, and the following parameters were collected and stored in an in-house study database: histological diagnosis and histotype of tumor, depth of myometrial invasion and involvement adjacent tissues, lymphvascular space invasion, age, BMI, menopausal status, menstrual status, HRT, type and duration of symptoms, date of diagnosis (matched), Stage of disease (FIGO I/II and III/IV, matched), grade, surgical procedure and its duration, blood loss and complications, lymphadenectomy including number of nodes removed, residual disease after primary surgery, chemotherapy regimen, number of cycles, response to treatment, time to progression, radiation treatment and date of death/relapse or last follow-up. Time to progression was defined as the time between the first day of treatment and either radiologic evidence of progressive disease or the first day of second-line treatment, whichever came first. Follow-up for all patients after treatment was at least every 3 months for the first 2 years, at least every 6 months for the subsequent 3 years and thereafter yearly for another 5 years or until death.

### Next-generation sequencing and immunohistochemistry

Due to the results retrieved from this retrospective analysis in MMMT-E, it was decided to continue along the lines also for MMMT-O. For this purpose, a prospective US-American cohort of 450 MMMT-O and 1290 high-grade serous ovarian cancers (HGSOC) which underwent immunohistochemistry (IHC) and next-generation sequencing (NGS) within a commercially CLIA-certified molecular profiling laboratory as referrals from 2008 to 2016 was used (Caris Life Sciences, Phoenix, AZ, USA). The tissue diagnoses were submitted based on pathological assessment of physicians who requested the assays and were further verified by a pathologist at the Caris Laboratory. IHC was performed on formalin-fixed paraffin-embedded tumor samples using commercially available detection kits, automated staining techniques (Benchmark XT, Ventana, Tucson, AZ, USA), antibodies against ERCC1 (Clone 8F1, Abcam), TUBB3 (Clone PRB-435P, BioLegend) and TOP2A (Clone 3F6, Leica Biosystems). ERCC1 loss was defined as ≤ 2 + staining in less than 50% of tumor cells or 3 + staining in less than 10% of tumor cells. TUBB3 loss was defined as less than 2 + staining present in less than 30% of tumor cells. A positive result for TOP2A was reported if at least 1 + staining was observed in 10% or more of tumor cells. Test results from the company’s commercial biomarker database were obtained anonymously using a data extraction tool.

### Statistical analyses

Descriptive statistics for study groups were presented using mean ± standard deviation (SD) or counts and percentages as appropriate. *P* values were calculated using T-tests or Fisher’s exact tests. Time to event was analyzed using Cox regression with corresponding hazard ratios (HR), 95% confidence intervals (CI), *P* values and the Kaplan–Meier method. A *P* value < 0.05 is considered as significant. Evaluations were done using the statistical software R version 3.1.1. Biomarker expression was compared across histologic subtypes via unpaired *t* tests using GraphPad software (GraphPad Software Inc, La Jolla, CA, USA).

## Results

We analyzed 103 histologically confirmed MMMT-E cases against 378 controls, consisting of 172 EC, 189 OC and 17 MMMT-O. The clinicopathological characteristics showed statistically significant differences for death from disease, age, BMI, FIGO Stage, grade, histotype, type of chemotherapy, adjuvant radiotherapy, residual disease, and lymph node dissection (*P *≤ 0.001, Table 1). Approximately, one-third of patients with MMMT-E (31.1%) and MMMT-O (35.5%) died due to their disease by the end of this study, in contrast to one tenth (9.9%) of EC and one-fifth (19%) of OC patients. Almost two-thirds (64.9%) in the MMMT-E study group and three quarters (76.4%) of the EC cases were early FIGO Stage I/II patients, in contrast MMMT-O (17.6%) and OC (32.4%). MMMT-O and OC patients were mainly advanced FIGO Stage III/IV cases (82.4% and 67.6%, respectively). Significantly more undifferentiated and high-grade cancers were found in MMMT-E and MMMT-O patients (82.2 and 93.3%, respectively) than in EC and OC patients (33.5% and 66.5%, respectively). Mainly, mixed and endometrioid histotypes were found in the MMMT-E study group (“other”, 71.4%) and in MMMT-O (55.6%) and EC (83.11%) patients; whereas OC patients were predominately of serous (66.7%). A total of 222 patients received adjuvant chemotherapy, hereby platinum and either different anthracyclines or ifosfamide were mainly given in MMMT-E (88.7%) and less in EC (31.6%), MMMT-O (42.7%) and OC (14.7%). In contrast, a platinum/taxane regimen was used most commonly in OC (85.3%), MMMT-O (57.1%), EC (68.7%) and only in 11.3% in MMMT-E. Adjuvant radiotherapy was applied in 65.3% to MMMT-E, 45.9% to EC, and 26.7% to OC.

**Table 1:** Clinicopathological characteristics of the Swiss/Australian cohort

	MMMT-E *N*=103 21.4%	EC *N*=172 35.8%	MMMT-O *N*=17 3.5%	OC *N*=189 39.3%	Overall *P* value	*N* 481
Age (years)						
Mean ± SD	68.8±11.2	66.6±11.8	68.2±12.0	63.3±12.9	0.001	481
BMI						
Mean ± SD	28.9±7.8	33.5±10.5	26.5±6.7	26.6±6.0	< 0.001	274
FIGO stage	94	165	17	185	< 0.001	461
Stage I/II	61 (64.9%)	126 (76.4%)	3 (17.6%)	60 (32.4%)		250
Stage III/IV	33 (35.1%)	39 (23.6%)	14 (82.4)	125 (67.6%)		211
Grade	45	164	15	161		385
Grade 3	37 (82.2%)	55 (33.5%)	14 (93.3%)	107 (66.5%)	< 0.001	213
Other	8 (17.8%)	109 (66.5%)	1 (6.7%)	54 (33.5%)		172
Histotype	35	172	9	188	<0.001	404
Serous	10 (28.6%)	29 (16.9%)	4 (44.4%)	126 (67.0%)		169
Other	25 (71.4%)	143 (83.1%)	5 (55.6%)	62 (33.0%)		235
Chemotherapy	53	19	14	136	< 0.001	222
P/A	47 (88.7%)	6 (31.6%)	6 (42.9%)	20 (14.7%)		79
P/T	6 (11.3%)	13 (68.4%)	8 (57.1%)	116 (85.3%)		143
Adjuvant RT	95	98	17	15	< 0.001	222
Yes	62 (65.3%)	45 (45.9%)	0 (0%)	4 (26.7%)		111
RD	71	62	16	9	< 0.001	158
None	59 (83.1%)	53 (85.5%)	7 (43.8%)	6 (66.7%)		122
LND	95	74	15	8	< 0.001	192
Any	66 (69.5%)	43 (58.1%)	2 (13.3%)	5 (62.5%)		116
n.d.	29 (30.5%)	31 (41.9%)	13 (86.7%)	3 (37.5%)		76
DOD	32 (31.1%)	17 (9.9%)	6 (35.3%)	36 (19.0%)	< 0.001	91

*EC* endometrial cancer, *MMMT-E* malignant mixed Mullerian tumors of the endometrium, *MMMT-O* malignant mixed Mullerian tumors of the ovary, *OC* ovarian cancer, *BMI* body mass index, *P/A* platinum/anthracycline, *P/T* platinum/taxol, *RT* radiotherapy, *RD* residual disease, *LND* lymph node dissection done (“any”) or not done (“n.d.”), *DOD* death of disease; statistical significance given by *P* values

**Fig. 1:**
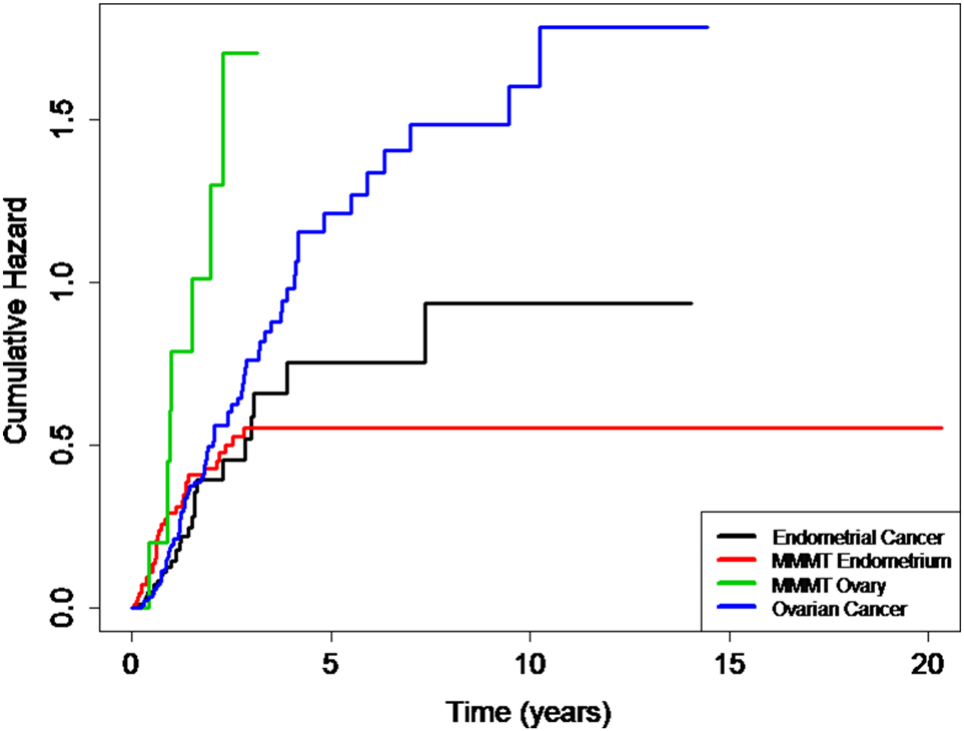
Cumulative 20-year risk for relapse in MMMT-E, EC, MMMT-O and OC in our cohort. After an initial increase within 2.5 years, the cumulative hazard for relapse remained constant for MMMT-E (red line), whereas it continuously increased for endometrial cancer (black line), ovarian cancer (blue), and MMMT-O (green) over the years

**Fig. 2:**
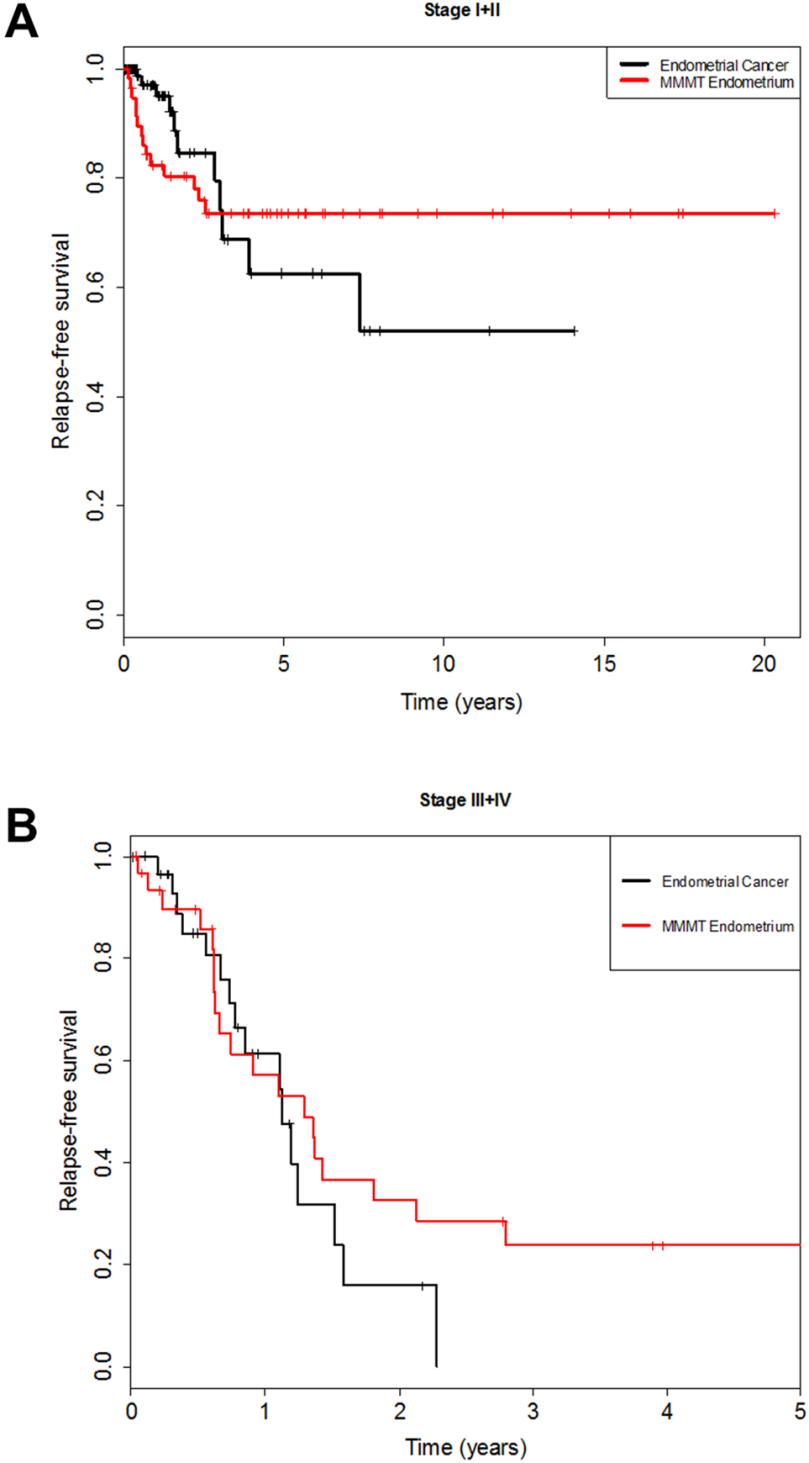
Kaplan–Meier curve comparing early Stage (FIGO I/II) and late Stage (FIGO III/IV) MMMT-E and EC patients. Relapse-free survival for **a** early Stage MMMT-E patients (red line) reached plateau at 2.5 years and remains constant until the end of the observation period (20 years), whereas it further decreased for the respective EC patients with time (observation period 14 years); and **b** late Stage MMMT-E reached a plateau after 2.75 years, meaning that almost a quarter of these patients did not relapse after this time until the end of the observation period (5 years), whereas all EC patients relapsed already after 2.25 years. Hence, the early FIGO Stage I/II MMMT-E patients account for the observed plateau after 2.5 years

We compared the long-term outcome, expressed as the cumulative risk of relapse, over a period of 20 years regardless of FIGO Stage. The cumulative relapse risk initially increased for all four cancers, strongest for MMMT-O patients and to comparable extents for OC, EC, and MMMT-E (Fig. 1). Intriguingly, however, the cumulative risk for MMMT-E patients remained stable, namely reaching a plateau after 2.5 years until the end of the observation period (20 years); whereas, it further increased for the three other cancers to differing extent over this time period. We speculated as to whether the observed divergent survival results for MMMT-E and EC was dependent on the FIGO Stage. The relapse-free survival of MMMT-E and EC were, therefore, compared for early Stage (FIGO I/II) (Fig. 2a) and late Stage (FIGO III/IV) (Fig. 2b) patients in a Kaplan–Meier presentation. Indeed, the relapse-free survival rate of early Stage MMMT-E patients decreased to a greater extent within the first 2.5 years when compared to EC patients, but then remained stable at 0.75, meaning that 75% of MMMT-E patients remained without any case of recurrence occurring for 20 years. The relapse-free survival rate of early FIGO stage EC patients was significantly different from that of the MMMT-E patients (strong intersection of the curves, test for proportional hazard *P *< 0.001) and decreased to a lesser extent than in MMMT-E patients within the first 2.5 years but then further decreased within the subsequent 11.5 years. The respective relapse-free survival rates for advanced FIGO Stage MMMT-E and EC patients decreased to a comparable extent (proportional hazard *P *= 0.118).

**Fig. 3:**
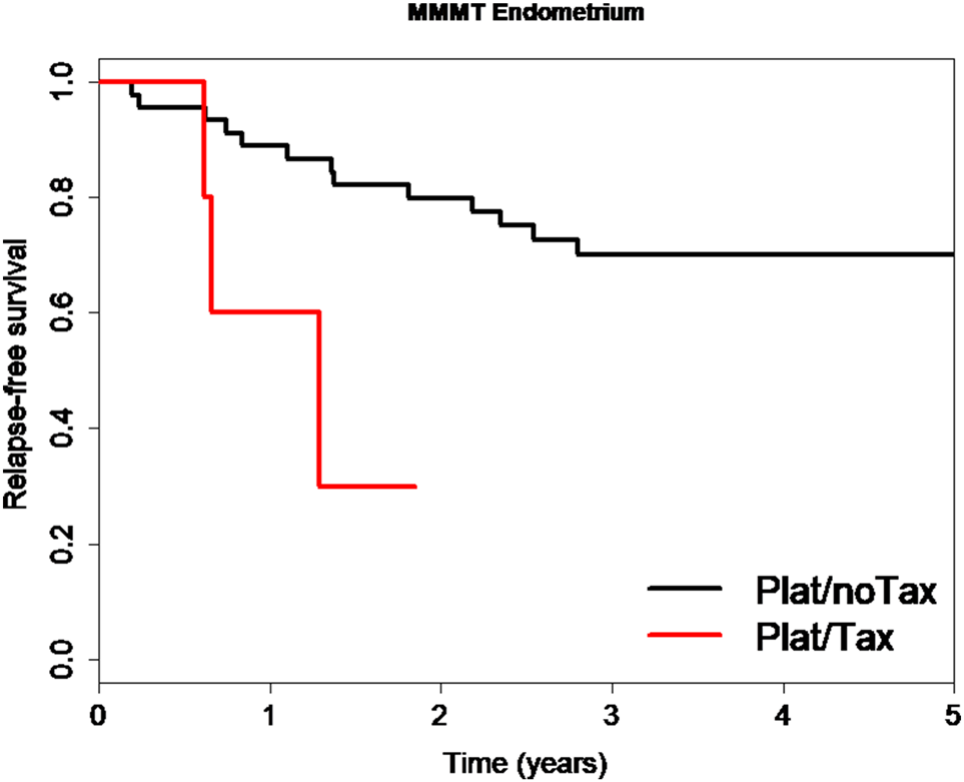
Kaplan-Meier curve comparing relapse-free survival in MMMT-E patients subjected to platinum chemotherapy with or without taxanes. MMMT-E patients receiving platinum and taxanes (Plat/Tax: red line) had a substantially worse relapse-free survival and shorter time to relapse (median: 1.29 years) than those with platinum and anthracyclines or ifosfamide (Plat/noTax: black line. Median time to relapse not available as median not reached) (HR 4.69, CI 1.23–17.87, *P *= 0.024)

We also analyzed whether the choice of the chemotherapy regimen affected the observed favorable relapse-free survival of MMMT-E patients, regardless of FIGO Stage. Indeed, a significantly shorter time to relapse for patients receiving platinum and taxanes than those receiving platinum and anthracyclines or ifosfamide was observed (HR 4.69, CI 1.23–17.87, *P *= 0.024, Fig. 3). This indicates that MMMT-E patients who received taxane-free platinum combination chemotherapy and particularly Stage FIGO I/II MMMT-E patients who do not relapse within the first 2.5 years have excellent long-term survival outcomes.

**Fig. 4:**
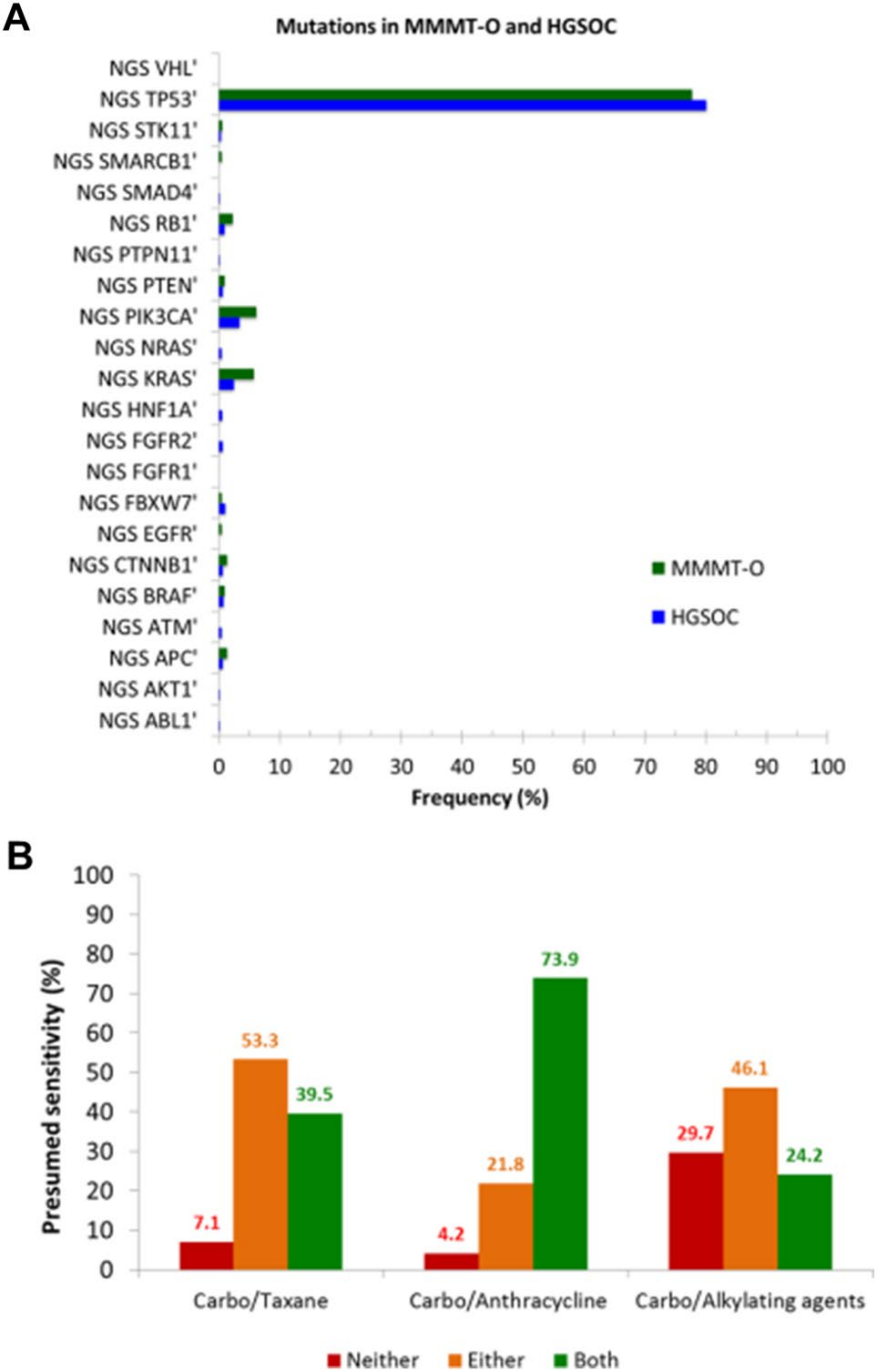
Mutational load in MMMT-O and HGSOC and expression of chemotherapy target proteins in MMMT-O. **a** NGS-data showing the mutational load (frequency expressed in %, *y*-axis) in MMMT-O (green) and HGSOC (blue). Mutation frequency expressed as percentage (*y*-axis) is plotted against selected gene mutations (*x*-axis). *KRAS* and *PIK3CA* mutations were significantly more frequent in MMMT-O than in HGSOC (*P *= 0.015 and *P *= 0.018, respectively). **b** Presumed chemosensitivity (expressed as percentage, *y*-axis) for MMMT-O based on the predictive biomarker expression for the combination of platinum with taxanes (left), with anthracyclines (middle), and with alkylating agents (right). Results of the prediction of sensitivity were based on whether none (red), one (orange) or both (green) of the biomarkers were expressed. Highest presumed sensitivity in MMMT-O was for the carboplatin/anthracycline combination (73.9%; green, middle)

The increased risk of relapse in MMMT-E patients receiving taxane-based chemotherapy prompted us to also examine whether a similar effect could be present in MMMT-O. We, therefore, examined the mutational load and the protein expression of various drug targets in a large independent US-American cohort of MMMT-O (*N *= 450). MMMT-O and HGSOC (*N* = 1290) displayed a similarly high load of *p53* mutations (77.8% vs 80.2%, Fig. 4a). In contrast, significant differences were found for *KRAS* and *PI3KCA* mutations, both being more frequent in MMMT-O compared to HGSOC (*KRAS:* 5.7% vs 2.4%, *P *= 0.015; *PI3KCA:* 6.2% vs 3.3%, *P *= 0.018).

The majority of proteins selected showed a statistically significant difference in the expression between MMMT-O and HGSOC (Supplement Table S1). These results, therefore, suggest that crizotinib (targeting ALK), cetuximab (EGFR), topotecan (TOPO1), anthracyclines (TOP2A), and alkylating agents such as ifosfamide (MGMT loss) would have a potential treatment benefit in MMMT-O. We next calculated a presumed sensitivity for the chemotherapy combination carboplatin/taxane compared to carboplatin/anthracycline or carboplatin/alkylating agents in MMMT-O. Hereby, the prediction of sensitivity to platinum/taxane combination was based on the loss of expression of ERCC1 and TUBB3 proteins, the prediction for the platinum/anthracycline combination was based on ERCC1 loss and TOP2A expression, and the prediction of platinum/alkylating agents combination was based on ERCC1 loss and MGMT loss. The results demonstrated for MMMT-O a presumed chemotherapy sensitivity of 39.5% for a combination of carboplatin/taxane, of 73.9% to a combination of carboplatin/anthracyclines, and of 24.2% to a combination of carboplatin alkylating agents (Fig. 4b). These data suggest that not only MMMT-E but also MMMT-O patients may benefit from a taxane-free chemotherapy.

## Discussion

It is increasingly important to define malignant diseases in relation to their genomic similarity instead of the organ of origin. This has been nicely shown in the M-PACT-trial from the US-National Cancer Institute, where the intention was to detect the molecular signature of diseases, rather than classify them by the tissue of origin. Subsequently, patient outcomes were examined according to mutations for *p53* and *PIK3CA*. Whilst the curves initially looked genetically driven, they diverged when the researchers looked at the tissue of origin (Schott et al. 2015).

MMMT of endometrial and ovarian origin share the same histological signature but their genetic similarity is still widely unknown. As MMMT-E and MMMT-O derive from different organs, they are not managed identical but due to the tissue of origin. One study suggests that MMMT-O and MMMT-E are different diseases in terms of their genetic landscape. In this study, 110 MMMT-O, 141 MMMT-E and 1587 OC of all histotypes where compared. *TP53* was the most commonly mutated gene in all three cancers with 76.4% in MMMT-O, 68.8% in MMMT-E and 69% in OC. Genetic alterations of *PI3K/AKT/mTOR* and *MAPK* pathways were noted to be similar in MMMT-O and OC but less frequent in MMMT-E (*p *< 0.001). In OC, the chance of having a BRCA1/2 mutation was highest compared to MMMT-E and MMMT-O (20% and 18% vs. 9%, respectively) (Mahdi et al. 2015). These data are similar to our own NGS analysis; however, we have 4-times more numbers of MMMT-O included and compared them to the most aggressive subtype of OC, HGSOC. Our own data in conclusion with the literature demonstrate a higher rate of *KRAS* and *PI3KCA* mutations in MMMT-O compared to HGSOC.

Since MMMT are known to be metaplastic carcinoma, they are no longer considered a subtype of sarcoma or managed as such. Instead, despite the lack of specific data, the management of MMMT has been extrapolated from studies of EC and OC (Berton-Rigaud et al. 2014; Cantrell et al. 2015). In the past 13 years, 9 GOG trials were performed in MMMT-E and MMMT-O. In total, 21 studies were found in our systematic literature search. Hereby, 16/21 were performed in MMMT-E only, 4/21 in MMMT-O only and 1/21 in both types (Table 2). In total, 1214 patients were included in these heterogeneous studies. The largest GOG trial incorporated 206 patients, but most studies examined MMMT numbers below 100, which clearly limits its results. Cisplatin-based chemotherapy and adjuvant setup were most commonly studied (67%), hereby the drug combination was mainly platinum plus paclitaxel or ifosfamide. Throughout these investigations, with mostly insufficient numbers of MMMT-E and MMMT-O patients, best response rate of a combination of carboplatin and paclitaxel was 62% and 55%, respectively, and 5-year overall survival 62–88% and 30%, respectively (Table 2). These data differ from our own results and might be due to the heterogeneity of the various cohorts and the small patient numbers in the published literature.

**Table 2:** Published studies on treatment modalities for MMMT-E and MMMT-O

Authors	Journal (year)	*N*	Design	Disease	Treatment	Outcome
Fowler, GOG Study Group	Gynecol Oncol (2002)	28	Prospective	MMMT-E Stage III/IV persistent or recurrent	Trimetrexate 5 mg/m^2^ b.i.d. for 5 days and repeated in 14 days	Overall RR 4.8%.
Duska	Gynecol Oncol (2002)	55	Retrospective	MMMT-O Stage II–IV	Carboplatin/Paclitaxel	Complete CR 55%; OS 27.1 months
Thipgen, GOG Study Group	Gynecol Oncol (2004)	136	Prospective	MMMT-O	Cisplatin (50 mg/m^2^) every 3 weeks until progression or toxicity	RR 20%, similar to MMMT-E
Sutton, GOG Study Group	Gynecol Oncol (2005)	76	Prospective	MMMT-E Stage I/II	Ifosfamide 1.5 g/m^2^ iv); Cisplatin 20 mg/m^2^	5-year OS 62%
Miller, GOG Study Group	Gynecol Oncol (2005)	51	Prospective	MMMT-E Persistent or recurrent	Phase II Topotecan 1.5 mg/m^2^ iv until progression or toxicity	No major activity
Homesley, GOG Study Group	JCO (2007)	179	Prospective	MMMT-E Stage III/IV persistent or recurrent	Phase III Ifosfamide 2.0 g/m^2^ iv or Ifosfamide 1.6 g/m^2^ iv and Paclitaxel 135 mg/m^2^	Median PFS and OS, combination treatment 3.6 v 5.8 v 3.6 and 13.5 v 8.4 months
Leiser	Gynecol Oncol (2007)	30	Retrospective	MMMT-O Stage II–IV	Platinum and Taxane	5-year OS 30%
Wolfson, GOG Study Group	Gynecol Oncol (2007)	206	Prospective	MMMT-E Stage I–IV	WAI or 3 cycles of Cisplatin and Ifosfamide	Chemotherapy arm lower RR 21% and death rate 29%
Makker	Gynecol Oncol (2008)	49	Retrospective	MMMT-E Stage I–IV	Paclitaxel–Carboplatin; Ifosfamide–Platinum; other CT; RT concurrent or alone	Paclitaxel–Carboplatin most efficacious
Signorelli	Int J Gynecol Cancer (2009)	41	Retrospective	MMMT-O	Cisplatin, Adriamycin, and Cyclophosphamide vs Cisplatin, Epirubicin, and Ifosfamide	Cisplatin, Adriamycin, and Cyclophosphamide:good RR but high toxicity
Hoskins	Gynecol Oncol (2008)	39	Prospective	MMMT-E	Paclitaxel 175 mg/m^2^, carboplatin (AUC 5-6) for 3-6 cycles ± radiation	RR 55–60%
Miller, GOG Study Group	Gynecol Oncol (2010)	28	Prospective	MMMT-E Persistent or recurrent	Phase II gemcitabine 600 mg/m^2^ and Docetaxel 35 mg/m^2^ iv days 1, 8 and 15 until progression or toxicity	Docetaxel and Gemcitabine not active
Galaal	Cochrane Database Syst Rev (2011)	579	Retrospective	MMMT-E Persistent or recurrent	RT and/or systemic chemotherapy	Chemotherapy with Ifosfamide and Paclitaxel should be considered
Lacour	Int J Gynecol Cander (2011)	23	Prospective	MMMT-E Persistent or recurrent	Phase II single arm 6 cycles of Carboplatin/Paclitaxel 3w	RR 62 %
Einstein	Gynecol Oncol (2012)	27	Prospective	MMMT-E	Ifosfamide (1.2 g/m^2^ and Cisplatin (20 mg/m^2^ vs Ifosfamide alone 3 cycles followed by pelvic external beam RT and brachytherapy followed by 3 additional cycles	No significant activity
Aghajanian, GOG Study Group	Gynecol Oncol (2012)	17	Prospective	MMMT-E	Paclitaxel 175 mg/m^2^ iv, Carboplatin AUC 6, Iniparib 4 mg/kg iv until disease progression or toxicity	No significant activity
Campos, GOG Study Group	Gynecol Oncol (2014)	22	Prospective	MMMT-E Recurrence	Phase II, second-line Pazopanib orally 800mg	Minimal activity
Lorusso	Int J Gynecol Cancer (2014)	46	Retrospective	MMMT-E Stage I–IV	Cisplatin 20 mg/m^2^ and Ifosfamide 1500 mg/m^2^ vs Carboplatin AUC 5 and Paclitaxel 175 mg/m^2^	Same efficiency but better toxicity profile with Carboplatin-Paclitaxel
Otsuki	Int J Gynecol Cancer (2015)	51	Prospective	MMMT-E Complete resection	Phase II single arm: 6 courses of 175 mg/m^2^ Paclitaxel and Carboplatin AUC 6	Combination of Paclitaxel and Carboplatin feasible and effective, 78.2% PFS, 87.9% OS
Vandenput	Int J Gynecol Cancer (2011)	69	Retrospective	MMMT-E and EC Stage I–I complete Staging	Platinum-based CT or no adjuvant therapy	RFS better with Platinum-based CT 22 vs. 10 months
Mackay	Gynecol Oncol (2012)	41	Prospective	MMMT-E MMMT-O Sarcoma Metastatic disease	Phase II VEGF TRAP (Aflibercept) Single Agent trial	Minimal activity

*GOG* Gynecologic Oncology Study Group, *RR* response rate, *OS* overall survival, *PFS* progression-free survival, *WAI* whole-abdominal irradiation, *CT* chemotherapy, *TR* radiation therapy, *AUC* area under the curve

Targeted drug trials have been scarce for MMMT with only two studies examining the role of VEGF-directed therapy, one with pazopanib (MMMT-E), the other with aflibercept (MMMT-E and MMMT-O), both demonstrating only a minimal efficacy (Campos et al. 2014; Mackay et al. 2012). Our large retrospective case–control study with over 20 years of follow-up has clearly shown that the combination of carboplatin/taxanes is less efficient in MMMT-E. Our exploratory data further suggest that the same could be the case for MMMT-O.

The concept of personalized treatment is based upon NGS, CISH and IHC data on potentially targetable biomarkers that describe an individual molecular footprint of a tumor (Janssens et al. 2017). Using precision IHC, we found proteins that were differentially expressed between MMMT-E and MMMT-O. It has been hypothesized that these markers could be associated with the likelihood of response to chemotherapy. If confirmed, this may explain the poorer performance of the current standard treatments. Obviously, this is only a hypothesis generating observation and a carefully designed prospective clinical trial would be necessary to validate these findings.

The rarity of MMMT, in particular of MMMT-O, accounts for the fact that subgroup analyses in randomized controlled chemotherapy trials can rarely be performed because of a lack of statistical power. Secondly, different histopathological definitions of MMMT make it difficult to compare studies that focus on this group. Thirdly, many reports combine MMMT-E and MMMT-O with their organ-based adenocarcinoma counterparts. Treatment options tailored to the mutational driver *p53* or *KRAS/PI3KCA* as shown in our NGS analysis should, therefore, be considered in future studies to tailor treatment in relation to the genetic origin. For this, collaborative studies within trial networks performing whole genomic sequencing of these tumors is needed to identify potential targeted therapies for the future.

## Electronic supplementary material

Below is the link to the electronic supplementary material.

Supplementary material 1 (DOCX 24 kb)
