# Supplementary material for: Should MMMT still be treated with adjuvant taxane-based combination chemotherapy?
Source: J Cancer Res Clin Oncol. 2020 Jan 28;146(3):695–704. doi: 10.1007/s00432-019-03091-y (PMC7039840; doi:10.1007/s00432-019-03091-y)
Supplement: Supplementary file 1 — Supplementary material 1 (DOCX 24 kb) [file 432_2019_3091_MOESM1_ESM.docx]

**Supplement Table S1 Immunohistochemical expression (in percentage) of selected protein targets in MMMT-O and HGSOC**

| **Target protein** | | **Expression (%)** | | |
| --- | --- | --- | --- | --- |
| **Short name** | **Full name** | **MMMT-O** | **HGSOC** | ***P*-value** |
| **ER** | Estrogen receptor | 9.3 | 47.5 | 0.0006 |
| **MGMT** | O6-Methylguanin-DNA-methyltransferase | 55.9 | 21.9 | <0.0001 |
| **RRMI** | Ribonucleotide reductase catalytic subunit M1 | 52 | 75.1 | <0.0001 |
| **AR** | Androgen Receptor | 20.4 | 43 | <0.0001 |
| **TUBB3** | Tubulin beta-3 chain | 47.4 | 66.4 | 0.0029 |
| **ALK** | Anaplastic lymphoma kinase | 27.3 | 9.2 | <0.0001 |
| **EGFR** | Epidermal growth factor receptor | 59.2 | 42.2 | 0.0066 |
| **TS** | Thymidylate synthase | 18.5 | 34.3 | 0.0062 |
| **PR** | Progesterone receptor | 21.1 | 36.8 | <0.0001 |
| **TOPO1** | Type I topoisomerase | 43.7 | 36.2 | <0.0001 |
| **TOP2A** | DNA topoisomerase II alpha | 85.6 | 79.4 | 0.028 |
| **PGP** | P-glycoprotein | 13 | 6.9 | 0.0004 |

Proteins are sorted in order of declining magnitude of difference in expression.
